# HCV elimination among people who inject drugs. Modelling pre- and post–WHO elimination era

**DOI:** 10.1371/journal.pone.0202109

**Published:** 2018-08-16

**Authors:** Ilias Gountas, Vana Sypsa, Sarah Blach, Homie Razavi, Angelos Hatzakis

**Affiliations:** 1 Department of Hygiene, Epidemiology & Medical Statistics, School of Medicine, National and Kapodistrian University of Athens, Athens, Greece; 2 Center for Disease Analysis, Lafayette, Colorado, United States of America; Centers for Disease Control and Prevention, UNITED STATES

## Abstract

**Background:**

Elimination of hepatitis C virus (HCV) among people who inject drugs (PWID) is a costly investment, so strategies should not only focus on eliminating the disease, but also on preventing disease resurgence. The aims of this study are to compute the minimum necessary antiviral therapies to achieve elimination with and without the additional expansion of harm reduction (HR) programs and to examine the sustainability of HCV elimination after 2030 if treatment is discontinued.

**Method:**

We considered two types of epidemic (with low (30%) and high (50%) proportion of PWID who engage in sharing equipment (sharers)) within three baseline chronic HCV (CHC) prevalence settings (30%, 45% and 60%), assuming a baseline HR coverage of 40%. We define sustainable elimination strategies, those that could maintain eliminations results for a decade (2031–2040), in the absence of additional treatment.

**Results:**

The model shows that the optimum elimination strategy is dependent on risk sharing behavior of the examined population. The necessary annual treatment coverage to achieve HCV elimination under 45% baseline CHC prevalence, without the simultaneous expansion of HR programs, ranges between 4.7–5.1%. Similarly, under 60% baseline CHC prevalence the needed treatment coverage varies from 9.0–10.5%. Increasing HR coverage from 40% to 75%, reduces the required treatment coverage by 6.5–9.8% and 11.0–15.0% under 45% or 60% CHC prevalence, respectively. In settings with ≤45% baseline CHC prevalence, expanding HR to 75% could prevent the disease from rebounding after elimination, irrespective of the type of the epidemic. In high chronic HCV prevalence, counseling interventions to reduce sharing are also needed to maintain the HCV incident cases in low levels.

**Conclusions:**

Harm reduction strategies have a vital role in HCV elimination strategy, as they reduce the required number of treatments to eliminate HCV and they provide sustainability after the elimination. The above underlines that HCV elimination strategies should be built upon the existing HR services, and argue for HR expansion in countries without services.

## Introduction

People who inject drugs (PWID) represent the core of the hepatitis C virus (HCV) epidemic in many high-income countries [1]. HCV is transmitted in this population predominantly by sharing contaminated needles and infusion equipment [1, 2]. Recently, significant advances in the antiviral treatment of HCV have improved the management of the infection, achieving high sustained viral response (SVR) rates (more than 90%) over a short duration of therapy (up to 12 weeks) [3, 4]. Due to the clinical achievements, global elimination of HCV is being seriously considered worldwide [5–9]. According to WHO, the elimination of HCV as public health problem includes the decrease in incidence by 80% and of HCV-related mortality by 65% by 2030, compared to 2015 [5]. One of the ways to achieve these targets in the population of PWID is to use antiviral treatment as prevention (TasP), to reduce the pool of infected and, subsequently, to prevent onward transmission [7, 10–12].

Numerous mathematical models have shown that HCV antiviral treatment is an efficient strategy to reduce chronic hepatitis C (CHC) prevalence and incidence among PWID [7, 8, 12–14]. In addition, harm reduction programs (HR) (like opioid substitution therapy and needle and syringe programs), constitute another effective way to prevent the spread of disease [8, 15]. When those interventions are combined, greater reductions in CHC prevalence and incidence could be achieved [8, 16]. However, it is important to take into consideration additional aspects of the high-risk behaviors e.g. the frequency of shared injections per person per year or the proportion of PWID who engage in sharing equipment (sharers), in order to design accurate models to guide healthcare interventions. For example, PWID who are not sharing their injection equipment are not at risk of HCV infection.

We used an extension of a previous HCV transmission model [8] that takes into account the proportion of sharers in the population as well as the median frequency of injections per person per year shared. Furthermore, we allowed the force of infection to be nonlinearly related to prevalence. The aims of this study were to: 1) Compute the minimum number of oral antiviral therapies that are necessary to achieve 80% reduction in the number of new HCV infections by 2030 in different representative settings; 2) Explore the contribution of harm reduction programs in HCV elimination strategy; and 3) Examine the necessary interventions to prevent HCV from rebounding after achieving elimination.

## Methods

### Description of the mathematical model

**Fig 1:**
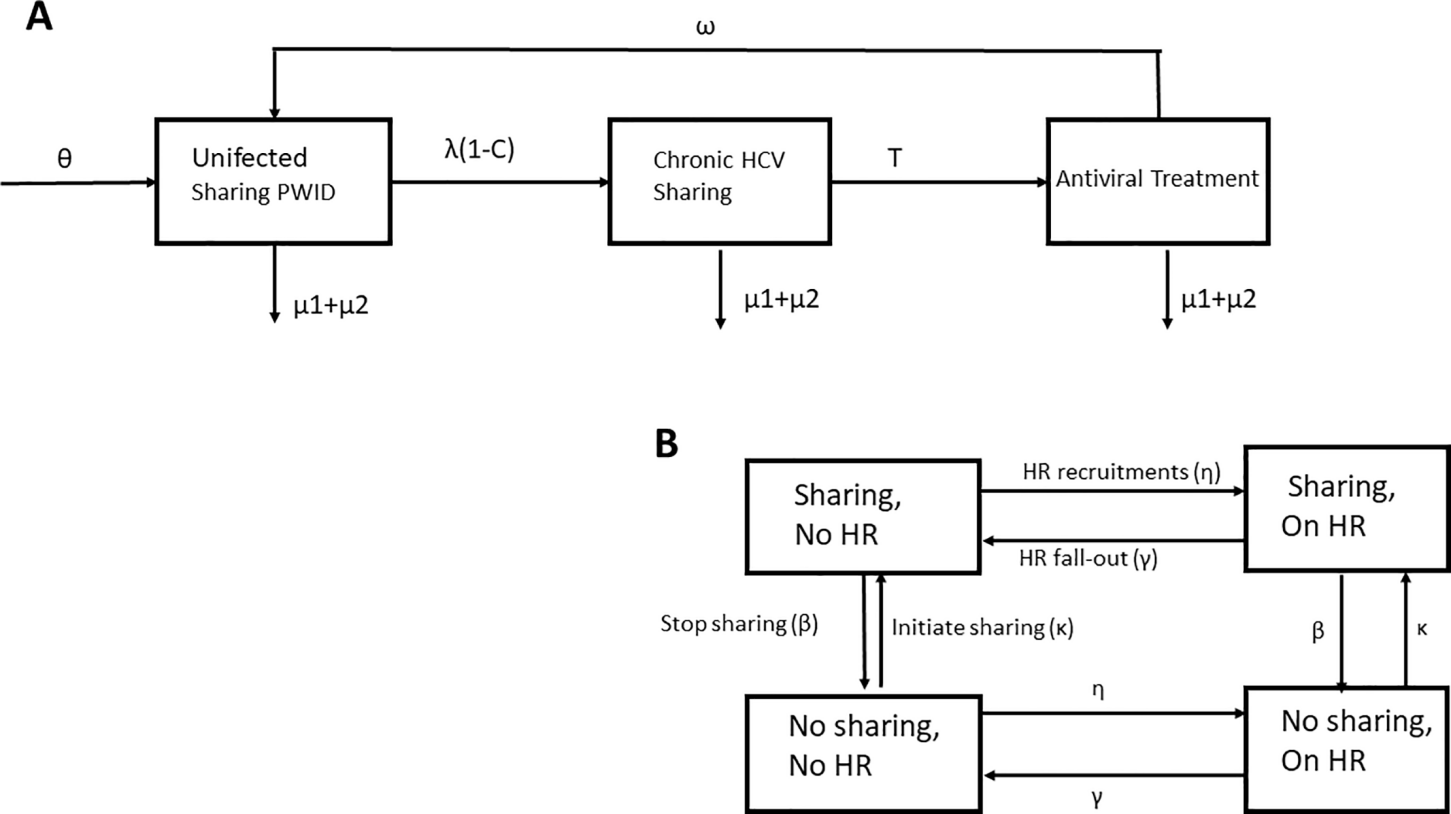
Schematic outline of the mathematical model for HCV disease transmission and treatment states (A) and behavioral states (B). Parameters: θ represent the new injectors flow rate for sharers and non-sharers, λ_i_ the infection rate per year i which depends on people who inject drugs (PWID) status (whether they participate in a harm reduction programs), C the proportion that spontaneously clears the infection, Τ the annual treatment rate, ω the proportion of PWID achieving sustained viral response, μ_1_ (mortality in the population of PWID), μ_2_ (rate of leaving PWID population due to injecting cessation).

A discrete time, stochastic, individual based model of HCV transmission among PWID was developed in C++ (v.5.6.3) with the model structure shown in Fig 1. The model follows transitions between three mutually exclusive compartments of PWID: 1) Susceptible PWID including those who either have never been HCV infected, have spontaneously cleared infection or have had successful treatment; 2) HCV-infected; and 3) PWID under treatment. The population of PWID was additionally stratified per sharing status (sharer or non-sharer), and whether the PWID participates in HR programs (Yes/No). The population of PWID was additionally stratified per sharing status (sharer or non-sharer), and whether PWID participate in HR programs (Yes/No). Initially, all new injectors are classified as sharers and not in a harm reduction program, given that there are data demonstrating that PWID are at higher risk of HCV infection during the first years of their injecting career [17, 18].

Each year, PWID exit through death (μ_1_) or cessation of injection (μ_2_) and enter to general population at rate θ, set equal to keep the population size at constant levels. We modeled the force of infection to depend on prevalence, and whether PWID are at sharing phase, participate in harm reduction program or both. More specifically, the force of infection for susceptible PWID participants in HR program is multiplied by a factor Ζ (Z<1) indicating that PWID in HR programs have lower probability of getting infected compared to PWID not in HR programs [19, 20]. We assumed that only sharers could be infected and that the proportion of sharer and non-sharer PWIDs remained constant over time. Potential natural immunity following successful treatment and transmission through sexual activity and non-injection drug use were not considered in the model. More details about the model can be found in the S1 File and S1 Table.

### Model parameterization

We examined three scenarios of HCV epidemics among PWID (30%, 45% and 60% CHC prevalence). Those epidemics were divided according to the prevalence of risk behaviors of the PWID population (low or high proportion of sharers). All 6 combinations of CHC prevalence and injecting risk behaviors were considered.

Specifically, we divided the 3 epidemics (30%, 45% and 60% CHC prevalence) in settings where PWID have low (30% sharers) or high (50% sharers) risky practices. In all the above examples, we assumed that the prevalence of HCV antibodies would be up to 26% higher than the prevalence of HCV RNA [21]. All the inputs are shown in Table 1. We considered the above combinations of CHC epidemics and risk behaviors, since they could be matched in several international settings (S2 Table). For example, a setting with low CHC prevalence and high proportion of sharers could be Belgium [22]. A setting with medium CHC prevalence with low proportion of sharers could be Switzerland [23] or Hamburg Germany [24, 25]. Concerning settings with medium CHC prevalence and high proportion of sharers could be Italy [26] or Norway [27]. An example of a setting high CHC prevalence and low proportion of sharers could be Portugal [28]. Finally, settings with high CHC prevalence and high percentages of sharers could be Athens Greece [8], or St. Petersburg, Russian Federation [29].

**Table 1:** Baseline model parameters.

Parameter	Estimate	Reference
Number of new people who inject drugs (PWID) per year	Fit a total population of 1000 PWID	
Duration of injecting career among PWID (1/μ_1_)	11 years	[31]
Probability of transmission of hepatitis C (HCV) from one contaminated injection	1.8%	[32]
Annual overall PWID mortality (μ_2_)	1%	[33]
Proportion of acutely infected spontaneously clearing infection (C)	26%	[21]
Proportion participating in harm reduction programs (β)	40%	[30]
Relative risk for HCV infection while in a harm reduction program (Z)	0.41	[19, 20]
Mean duration of harm reduction	2 years	[25]
Relative risk for HCV re-infection after counseling intervention (vs. without counseling)	0.7	[34]
Proportion achieving sustained virological response (SVR) under IFN-free DAAs	90% until 2017, 95% after 2018	[35, 36]

In our projections, we assumed medium baseline HR coverage of 40%. Although the coverage of harm reduction interventions is variable within the population of PWID, recent estimates have shown that about 40% of the total number of problem opioid users in EU participate in a harm reduction program [30]. In S3 Table and S7 Fig of the manuscript, scenarios concerning lower baseline HR reduction settings (e.g. baseline HR coverage of 20%) could be found.

### Modelled scenarios

We tested interventions of increasing treatment coverage or/and expanding HR coverage, and assessed their impact on the prevalence and incidence of HCV. We computed the minimum necessary treatment coverage to achieve WHO’s elimination goals concerning incidence (i.e. 80% reduction in HCV incidence in 2030 compared to 2017) without increasing HR coverage (40% baseline HR coverage) or with gradually expansion of HR coverage to 75% (about 3% per year) by 2030. We also examined the additional effect of education/mental health counseling interventions during treatment to reduce re-infection, in the treatment and HR scenario [34].

After achieving elimination, we halted treatment intervention and observed which strategies were capable of maintaining the elimination effects and preventing the disease from rebounding in the next 10 years (2031–2040). For each scenario, 1000 runs were performed and the results were summarized. In order to include the appropriate uncertainty (stochastic variability), the 2.5 and 97.5 percentiles of the simulations were also shown.

### Sensitivity analysis

To examine the impact of different model assumptions in the required treatments to achieve elimination, we undertook a univariate sensitivity analysis in the scenario of 45% prevalence with 50% sharers PWID. We explored the impact of shorter/longer average duration of injecting carrier (5 or 15 versus 11 years), the influence of changes in risk behavior after successful treatment (50% lower/higher probability of re-infection vs. no change in risk behavior), the impact of adherence to treatment in SVR (80%/100% vs. 90%-95%),the influence of greater or lesser difference in risk between PWIDs in HR programs vs. those who are not (0.2/0.8 vs. 0.41) and the impact of treatment prioritization for PWID who engaging in HR programs versus those who do not (70/30% vs. 50/50%).

## Results

### HCV elimination under various scenarios considering annual treatment coverage and harm reduction coverage

In settings with 30% chronic HCV prevalence, low annual treatment coverage (ranging between 2.7–3.0% (e.g. 27–30 annual treated PWIDs/ per 1000 PWIDs) dependent of the risk behaviors of the population) are needed in order to eliminate the disease in 2030, without additional expansion of HR coverage. Increasing HR coverage to 75% in 2030, had a minimal impact in reducing the required treatment coverage (reduction ranges between 3.5–6.5%), highlighting that in settings with low baseline prevalence, an increase in antiviral treatment coverage maybe in higher priority compared to the increase of HR coverage (S1 Fig).

**Fig 2:**
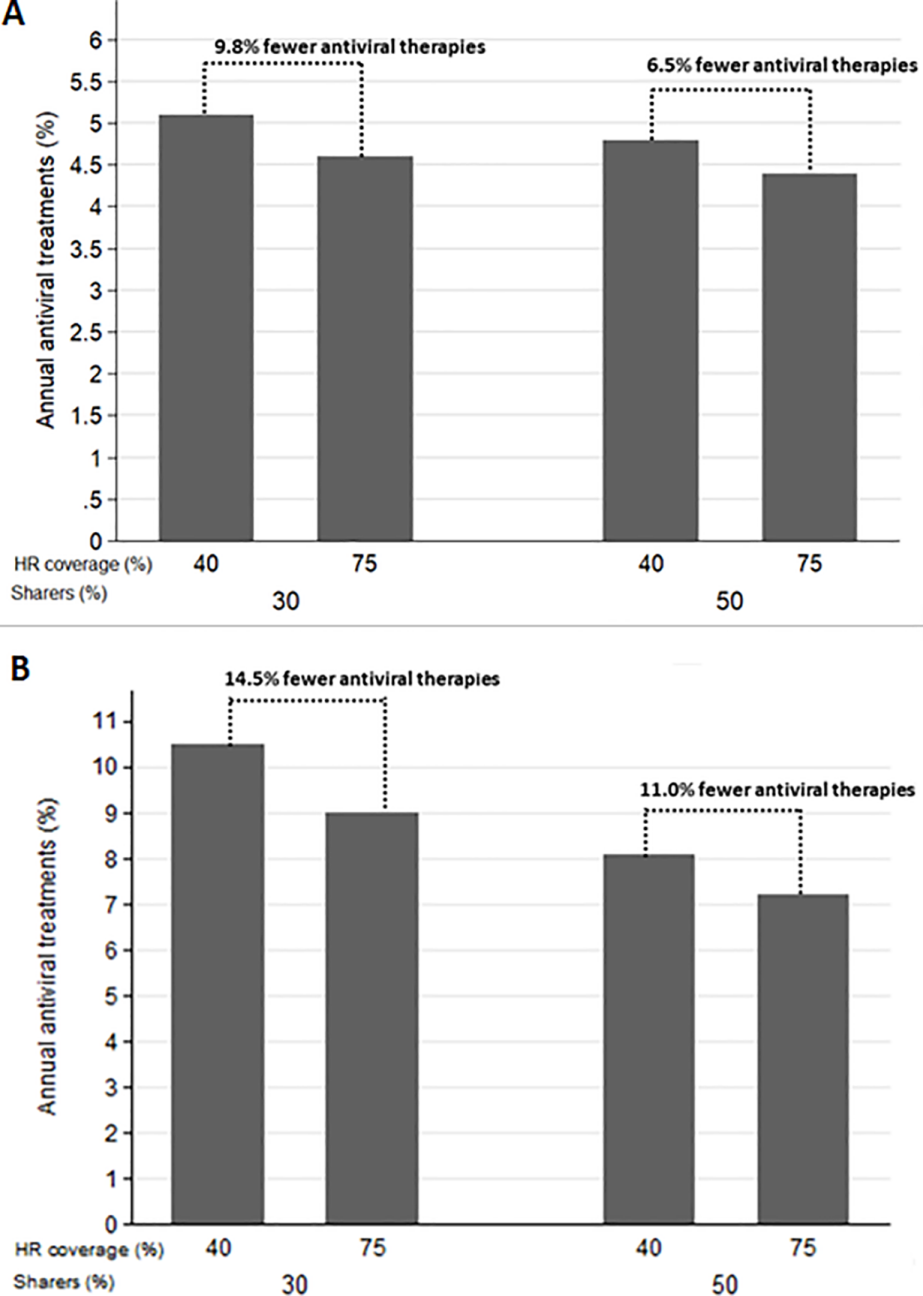
The needed treatment rate coverage (%) to achieve HCV elimination among PWID by 2030 under a in settings with 45% or 60% chronic hepatitis C prevalence. The bars correspond to scenarios of harm reduction coverage at 40% and 75%. A. 45% CHC prevalence B. 60% CHC prevalence.

In areas with 45% CHC prevalence, the needed annual treatment coverage to achieve elimination, without expansion HR ranges between 4.7–5.1% (e.g. 47–51 treated PWIDs/1000 PWIDs). Increasing HR coverage in those settings has higher impact, compared to an area with low CHC prevalence. In these settings expanding HR coverage by 3% per year until 2030 reduces the required treatment coverage by 6.5–9.8% (Fig 2A).

In areas with high baseline chronic HCV prevalence, the needed antiviral treatment rates varied significantly from 8.0–10.5% (Fig 2B). It is important that as the prevalence increases, the influence of the risky behaviors in the needed treatment coverage increases too. For example, the range of the required treatments in a setting with 30% or 45% baseline chronic HCV prevalence is narrower compared to a setting with 60% baseline chronic HCV prevalence. Furthermore, in these prevalence settings, the contribution of HR is high; expansion of HR coverage from 40% to 75% reduces the needed treatment number by 11–15%.

To achieve the WHO elimination targets it is vital to implement awareness or screening campaigns in order to create enough demand for treatment. If awareness or screening campaigns failed to diagnose enough patients and treat patients started to drop before 2030, WHO elimination targets are unlikely to be reached (S8 Fig).

The above results underline that different epidemics, require different types of intervention. For example, the effect of HR is more pronounced in environments with higher prevalence of risk behaviors. Therefore, in areas where the additional gains from expanding HR is limited, it may be better to implement strategies that mainly focus on antiviral treatment.

### Interventions to prevent the rebound of the disease after HCV elimination

Under low (30% baseline CHC prevalence) (data not shown) or medium prevalence settings (45% baseline CHC prevalence), treatment combined with 3% annual increase in HR coverage sufficiently prevented the rebound of HCV infections after 2030 regardless of the structure of the epidemic (proportion of sharers and number of unsafe injections) (S2 and S3 Figs). In these cases, the average time from first injection to HCV acquisition, under treatment-only scenario after 2030 is about 20 years.

**Fig 3:**
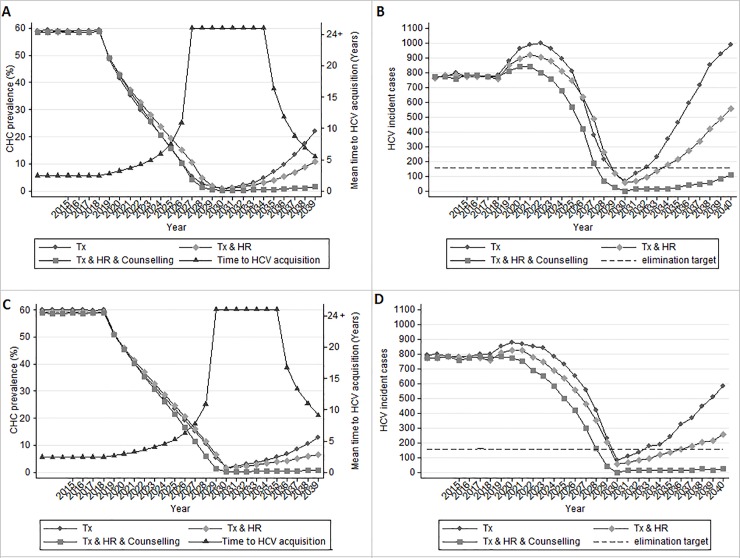
Model predictions assuming 60% chronic HCV prevalence and 30% or 50% of the people who inject drugs sharing injection equipment. We assumed that no treatment is provided after 2030.Mean time of HCV acquisition greater than 25 years is shown as 24+ years. Mean time corresponds to Tx only scenario. Tx: Antiviral treatment, HR: harm reduction, Counselling: Psychological interventions to reduce re-infections post- treatment. A. 30% sharers-Prevalence B. 30% sharers-HCV Incident cases C. 50% sharers-Prevalence D. 50% sharers-HCV Incident cases.

In Fig 3, estimates of HCV prevalence and of the number of new HCV infections are presented according to various strategies implemented post-elimination under a high HCV prevalence setting (60% CHC prevalence). In high prevalence settings HR alone would be insufficient to maintain the results of elimination. In those settings, to preserve the results of elimination, counseling interventions need to be implemented in addition to HR and treatment, to prevent the disease from rebounding. An alternative strategy to prevent the recovery of the disease is to lower the number of incident cases more than the proposed target of WHO (i.e. more than 80% reduction in 2030 compared to 2017) (S4 Fig).

### Sensitivity analysis

**Fig 4:**
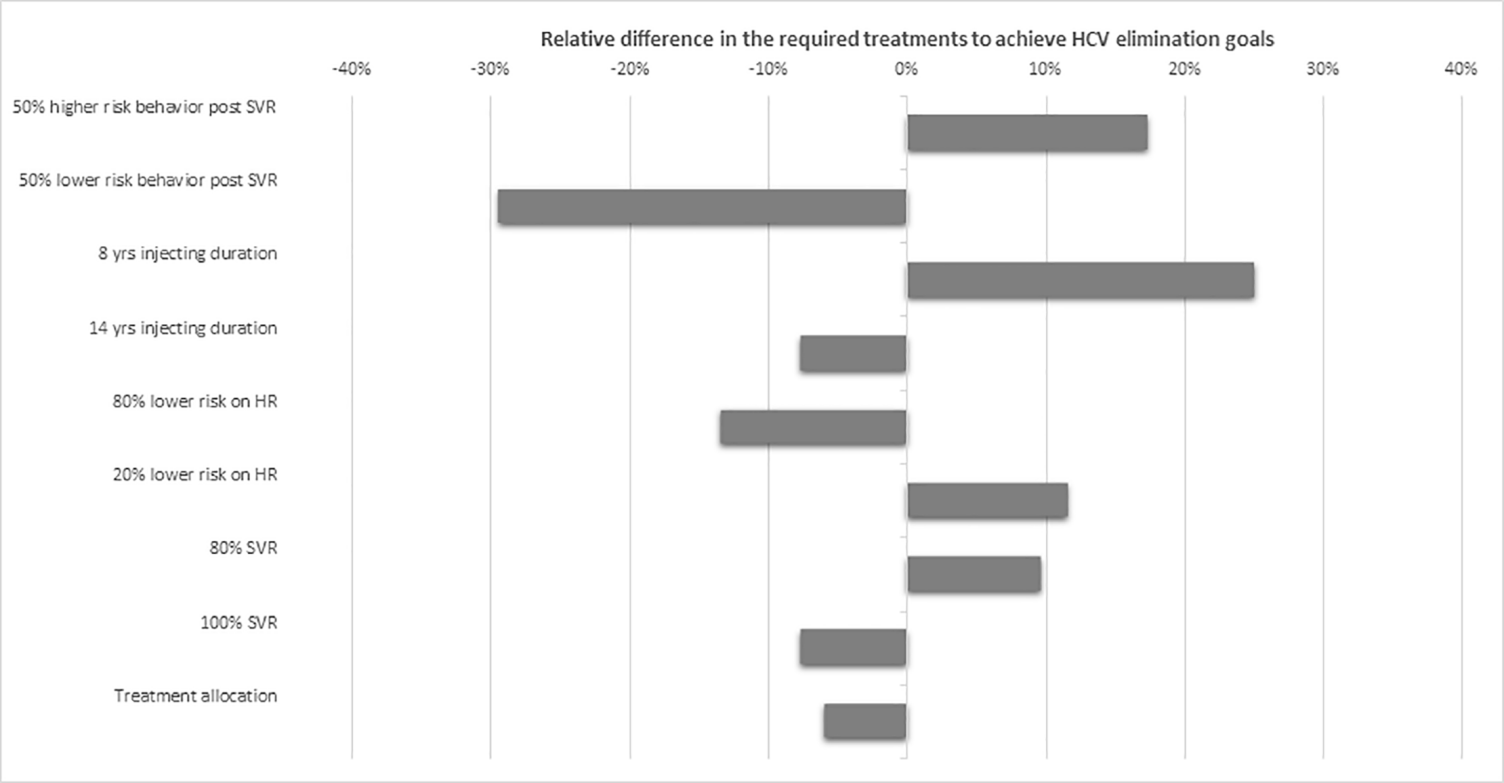
Results of one-way sensitivity analysis showing the relative difference in needed treatment to achieve HCV elimination goals for varying parameters of the model compared to the base parameter values in Table 1. A value of zero describes no change from estimated needed treatments compared to the base scenario. A positive or a negative value means that the required treatments are higher or lower to the estimated under the base scenario. The scenario of 45% chronic hepatitis C prevalence with 50% sharers was used.

According to sensitivity analysis, variations in duration of injecting drug use would affect the annual treatment coverage required to eliminate the disease. Specifically, under shorter injecting duration (8 years instead of 11 years) the required treatment coverage increased by 25% compared to the base case, while for longer injection duration (14 years instead of 11 years) treatment coverage decreased by 8% compared to the base case (Fig 4).

Variability in SVR as consequence of treatment adherence would affect the number of needed treatments. If SVR was 80% (instead of 90%-95% years), needed treatments would increase by 9.7% to eliminate the disease. On the other hand, if SVR was 100%, the treatments would decrease by 7.0% (Fig 4). Potential changes in risk behaviors post successful treatment also affected the model’s predictions. Specifically, if risk behaviors halved after treatment, the required treatments would be reduced by 26.4% compared to the base case (Fig 4). On the other hand, if risk behaviors increased by 50% after a successful treatment (e.g. due to complacency caused by the high efficacy of DAAs) the required treatment coverage would increase by 17.4%.

## Discussion

Elimination refers to the reduction of the incidence of the infection to near zero in a defined geographical area as a result of deliberate efforts but requires the presence of continued measures to prevent re-establishment of transmission. Elimination of HCV is a costly investment, so strategies should focus not only on eliminating the disease but also on preventing disease resurgence. This study, using an extended HCV transmission model, computes the necessary treatment coverage to achieve WHO’s elimination goals among PWID and examines which level of HR coverage could make HCV elimination sustainable i.e. to prevent disease from rebounding after HCV elimination. Reducing the prevalence of HCV contributes to the increasing of the average time to HCV acquisition. An indicator of the success of HCV elimination strategy may be to increase the average HCV acquisition time since first infection so that it is greater than the mean injecting duration of PWID. Using combined interventions to eliminate HCV, the benefits are maximized as HR programs reduce directly the incidence while antiviral treatment reduces directly the prevalence -and indirectly the incidence- of HCV infection.

According to our model, HCV elimination necessitates variable treatment coverage and significant improvements in primary prevention. More specifically, our results emphasize that the required treatments to achieve elimination strongly rely on baseline HCV prevalence as well as the population structure. It is more difficult to eliminate the disease in an environment where a small proportion of the PWID has a lot of unsafe injections, compared to a setting where a higher proportion of the population has fewer unsafe injections, under the assumption of totally mixed population. Therefore, it is important to consider the sharing behavior (injecting frequency and proportion of sharers) before implementing an HCV elimination strategy in this population. The effect of injecting frequency has been also underlined in network models of HCV transmission [37].

Harm reduction strategies have a vital and multifaceted role in reaching the HCV elimination goal, as the effect of treatment alone as prevention strategy fades as the baseline HCV prevalence increases. First, harm reduction reduces both initial infections and re-infection after successful treatments and, thus, the required treatments to eliminate HCV. We have shown that the effect of HR is more pronounced under high HCV prevalence settings. Second, HR strategies post elimination is an intervention which could maintain the results of treatment and prevent the disease from rebounding. It is important to note that in high prevalence settings, elimination is not sustainable, and is only feasible under combined interventions. Third, HR services could have a potential case-finding role and serve as an access point to HCV education and counseling. Its effects could be intensified by psychosocial/educational interventions aimed to reduce sharing and reinfections. All the above underline that HCV elimination strategies should be built upon the existing HR services, and argue for HR expansion in countries and settings without services.

In our analysis, we assumed that treatments would be discontinued after the elimination of HCV in 2030. This is definitely the worst-case scenario; it was used to emphasize that without the simultaneous expansion of HR, the epidemic may start recovering due to low treatment coverage. Treatment coverage may be reduced because of the low prevalence of viremic infection, difficulties to identify the last infected individuals, competing healthcare budget allocation priorities or potential complacency after achieving the goals of HCV elimination.

Re-infection is a barrier to implement treatment as prevention as an intervention to end transmission [38, 39]. A recent analysis [39] found that the overall pooled estimate of re-infection was similar to that of initial infection. Thus, in settings where background HCV infection was rare, the risk of re-infection was low. On the other hand, in settings where HCV prevalence is high the risk of re-infection would be high as well. In this paper, in line with other empirical and modeling studies [7, 10, 12, 16], we have assumed that the risk of re-infection was equal to that of initial infection. It is important that low or moderate treatment coverage would have a marginal effect on incidence, due to potential re-infections [8]. As we treat few PWID, without applying any additional interventions, the susceptible population would greatly increase and thus, the incidence could increase. However, the existence of re-infections is a sign that we treat the right population, which is the active PWID.

A significant uncertainty exists in whether the high clinical efficacy of DAAs (more than 90%) could be achieved in real-world settings and how potential changes in high risk behavior after successful treatment could impact the projections. In line with other viral illnesses, poor adherence results in lower likelihood of treatment response. Our sensitivity analysis showed that lower SVR (80% vs. 90–95%) would decrease the impact of the intervention and increase the number of needed treatment by almost 10% (Fig 4). On the other hand, if the risk of reinfection post treatment is 50% lower than the primary infection risk, the required treatments would be 26% lower, compared to base case. Therefore, treatment should be coupled with adherence and risk reduction counseling interventions.

The results of the models are also sensitive to the uncertainty about PWID risk behaviors. Specifically, the duration of injecting career caused the most uncertainty to the projections. According to the sensitivity analysis, under shorter injecting duration, i.e. in settings where the population of PWID is renewed more rapidly, the effect of treatments would shrink due to less time to accrue treatment benefits. On the contrary, if injecting career was longer than 11 years, the impact of treatment would be improved. Fundamental uncertainty about PWID behaviors still exists, which should be explored to limit the model's uncertainty.

### Added value of the study

Our study contributes to the discussion concerning the feasibility of HCV elimination in the key population of PWID. First, we underlined that harm reduction strategies have a vital role in the elimination strategy, not only to reduce the required treatments to eliminate HCV but also to provide sustainability in the post-elimination era. It is important that in high prevalence settings elimination of HCV is not sustainable if treatment would be halted, even if we simultaneously increase the coverage of harm reduction. In those settings either we should reduce incident cases more than 80% or we should apply counseling intervention post-successful treatment to reduce re-infection. Second, we highlighted that an increasing number of HCV re-infections will be expected in the initial years of DAA treatment scale-up, as a result of the expansion of the susceptible population. However, sustained HCV treatment strategies will reduce the infected pool leading to the eventual reduction in the rate of HCV re-infection. Third, we have shown that different epidemics require different types of intervention, thus, from health care resource allocation perspective, in settings with low baseline prevalence where the additional gains from expanding HR is limited, it may be better to implement strategies mainly focused on antiviral treatment.

### Comparison with other studies

Our model computes similar baseline incidence rates compared to previous mathematical modeling models [40]. Furthermore, our results are in line with a previous modeling studies showing that combination prevention with DAA therapy and harm reduction could have a substantial impact on chronic HCV prevalence and incidence among PWID populations [8, 16, 41, 42]. Finally, in our model we allow retreatment of reinfection to achieve elimination targets. The significance of retreatment of reinfections has been emphasized a recent modeling study applied in the U.S. [42].

### Limitations

Our analysis has some limitations. First, the model ignores the impact of social networks on HCV transmission and assumes that the population is totally mixed i.e. sharing injectors have equal contact with all other sharing injectors in the population. Second, we assumed that the proportion of sharer and non-sharer PWID remained constant over time after 2016. Third, HIV/HCV co-infections were not accounted for. The effect of coinfection on our projections is likely to be marginal as HIV/HCV coinfected PWID achieve similar SVR to HCV monoinfected PWID under DAA therapy [43]. However, in settings whereas the coverage of antiretroviral therapy for HIV infected PWID is low, the additional mortality derived from HIV may affect the results. Fourth, the model does not consider issues related to identifying HCV-infected PWID. Finally, issues like estimation of the cost of elimination or cost of the sustainability of the elimination of the HCV did not addressed in this paper. Future works examining the required cost to make HCV elimination sustainable are needed.

## Conclusion

In conclusion, our study demonstrates that HCV among PWID could be eliminated even in high prevalence and high-risk behavior settings. Using integrated healthcare strategies, the necessary treatment coverage to eliminate HCV is reduced and a post-elimination rebound of HCV could be prevented. Applying counseling interventions in addition to harm reduction and antiviral treatment would decrease re-infections and would prevent the disease from rebounding even in high risk settings.

## Supporting information

S1 File HCV elimination among PWID. (PDF)

S1 Table Proportion of sharers and number of injections per person per year. The number of injections is computed by the model in order to achieve the target of chronic hepatitis C prevalence given the proportion of sharers.
(PDF)

S2 Table Real life settings for each examined scenario of the manuscript. (PDF)

S3 Table Proportion of sharers and number of injections per person per year according to baseline HR coverage. The number of injections is computed by the model in order to achieve the target of chronic hepatitis C prevalence given the proportion of sharers.
(PDF)

S1 Fig Needed treatment coverage (%) to achieve HCV elimination by 2030 under a 30% chronic hepatitis C prevalence. The bars correspond to scenarios of harm reduction coverage at 40%, or 75% of PWID.
(PDF)

S2 Fig Model predictions concerning a 45% chronic HCV prevalence in which 30% of the PWID are sharers. We assumed that no treatments are given after 2030. Mean time of HCV acquisition greater than 25 years is shown as 24+ years. Mean time corresponds to Tx only scenario. Tx: Antiviral treatment, HR: harm reduction, Counselling: Psychological interventions to reduce re-infections post- treatment. A. Chronic HCV Prevalence, B. HCV Incident cases.
(PDF)

S3 Fig Model predictions concerning a 45% chronic HCV prevalence in which 50% of the PWID are sharers. We assumed that no treatments are given after 2030. Mean time of HCV acquisition greater than 25 years is shown as 24+ years. Mean time corresponds to Tx only scenario. Tx: Antiviral treatment, HR: harm reduction, Counselling: Psychological interventions to reduce re-infections post- treatment. A. Chronic HCV Prevalence, B. HCV Incident cases.
(PDF)

S4 Fig Model predictions concerning a 60% chronic HCV prevalence and 50% of the people who inject drugs sharing injection equipment. Tx: Antiviral treatment, HR: harm reduction. A. Sustainability of the elimination targets if incident cases reduced more than WHO elimination goals (e.g. 90% reduction in 2030 vs. to 80% reduction in 2030 compared to 2017).
(PDF)

S5 Fig Model predictions concerning a 45% chronic HCV prevalence in which 30% of the PWID are sharers. We assumed that no treatments are given after 2030. Tx: Antiviral treatment, HR: harm reduction. Model projection containing confidence intervals.
(PDF)

S6 Fig Model predictions concerning a 60% chronic HCV prevalence in which 30% of the PWID are sharers. We assumed that no treatments are given after 2030. Tx: Antiviral treatment, HR: harm reduction. Model projection containing confidence intervals.
(PDF)

S7 Fig Needed treatment coverage (%) to achieve HCV elimination by 2030 under a 60% chronic hepatitis C prevalence with 20% or 40% baseline Harm reduction coverage. (PDF)

S8 Fig Model predictions concerning a 45% chronic HCV prevalence in which 50% of the PWID are sharers. We assumed that treatments are reduced after 2020. Model projection assuming a decreasing of DAAs coverage prior to 2030 as a result of as a result of the unsuccessful the implementation of awareness or screening campaigns.
(PDF)
