# Supplementary material for: HCV elimination among people who inject drugs. Modelling pre- and post–WHO elimination era
Source: PLoS One. 2018 Aug 16;13(8):e0202109. doi: 10.1371/journal.pone.0202109 (PMC6095544; doi:10.1371/journal.pone.0202109)
Supplement: S1 File — (PDF) [file pone.0202109.s001.pdf]

Supplementary Materials

1) Probability of Infection

The probability of infection for an uninfected sharer PWID from *m* unsafe injections is a non-linear function of the probability of transmission of HCV from one contaminated syringe, the number of unsafe injections (*m*), and the prevalence of HCV-infected syringes. It is derived as follows:

$$\text{Probability}_{\text{Infection from } m \text{ Unsafe Injections}} = 1 - \text{Probability}_{\text{Not Getting Infected from } m \text{ Unsafe Injections}} \quad \text{(i)}$$

$$\text{Probability}_{\text{Not Getting Infected from } m \text{ Unsafe Injections}} = \left(1 - \text{Probability}_{\text{Infection from One Unsafe Injection}}\right)^{m} \quad \text{(ii)}$$

$$\text{Probability}_{\text{Infection from One Unsafe Injection}} = \text{Probability}_{\text{Transmission from One Contaminated Syringe}} \times \text{Prevalence}_{\text{Infected Syringes}} \quad \text{(iii)}$$

Combining (i), (ii), and (iii):

$$\text{Probability}_{\text{Infection from } m \text{ Unsafe Injections}} = 1 - \left(1 - \text{Probability}_{\text{Transmission from One Contaminated Syringe}} \times \text{Prevalence}_{\text{Infected Syringes}}\right)^{m} \quad \text{(iv)}$$

Furthermore, assuming that the rates of lending out and borrowing syringes among PWID are equal,

$$\text{Prevalence}_{\text{Infected Syringes}} = \frac{\sum_i \text{Unsafe Injections}_i \times \text{Fraction of PWID}_i \times \text{Prevalence of HCV Among Sharers}_i}{\sum_i \text{Unsafe Injections}_i \times \text{Fraction of PWID}_i}, \quad \text{(v)}$$

where *i* ranges over general population, harm reduction and fraction of PWID represents the proportion of PWID in group *i* *(1)*.

Specifically, for uninfected sharer PWID in group *i* (where *i* is general population, or on Harm Reduction), the probability of infection λ_i is:

$$\lambda = 1 - \left(1 - \text{Probability}_{\text{Transmission from One Contaminated Syringe}} \times \text{Prevalence}_{\text{Infected Syringes}}\right)^{m} \quad \text{(vi)}$$

The mean time to HCV acquisition was calculated by taking the reciprocal of probability of infection as follows:

$$Time\ to\ HCV\ acquisition = \frac{1}{\text{Yearly Probability of Infection}}$$

2) Model equations

$$\frac{dS}{dt} = -\lambda_{ij} * S * \left(\frac{I}{N}\right) - \left(\mu_1 + \mu_2\right) * S \ + \theta$$

$$\frac{dI}{dt} = \lambda_{ij} * S * \left(\frac{I}{N}\right) - I * Tr - \left(\mu_1 + \mu_2\right) * I$$

$$\frac{dT}{dt} = I * Tr - \omega * T \ - \left(\mu_1 + \mu_2\right) * T$$

Where:

$$\lambda_{ij} \begin{cases} \lambda\ , & if \quad Sharer = 1\ and\ HR = 0\ \ and\ Counseling = 0 \\ \lambda * C, & if\ sharer = 1\ and\ HR = 0\ and\ Counsling =\ 1 \\ 0\ , & if\ sharer = 0 \\ \lambda * Z, & if\ sharer = 1\ and\ HR = 1\ and\ Counseling = 0 \\ \lambda * Z * C\ , & if\ sharer = 1\ and\ HR = 1\ \ and\ Counseling = 1 \end{cases}$$

λ_{ij}: the force of infection where i= the HR status and j= the Counseling status

Tr: Treatment coverage

ω=SVR rate

In order to keep the overall population constant we set $\theta=\mu1+\mu2$

3) Description of the Mathematical Model

A discrete time, stochastic, individual based model of HCV transmission among PWID was developed in C++ (v.5.6.3) with the model structure shown in Figure 1 of the main text. The model follows transitions between three mutually exclusive compartments of PWID: 1) susceptible people including those who either have never been HCV infected, have spontaneously cleared infection or have had successful treatment; 2) HCV-infected; and 3) PWID under treatment. The population of PWID was additionally stratified per sharing status (sharer or non-sharer), and whether the PWID participates in HR programs (Yes/No). Initially, all new injectors are not participating in harm reduction programs.

Each year, PWID exit through death (μ_1) or cessation of injection (μ_2) and enter to general population (PWID not in harm reduction programs) at rates θ_1 (uninfected sharers) or θ_2 (uninfected non-sharers), equal to keep the population size at constant levels.

We model the force of infection, for uninfected sharer PWID, to be a non-linear function of the prevalence as shown above:

$$\lambda = 1 - \left(1 - \text{Probability}_{\text{Transmission from One Contaminated Syringe}} \times \text{Prevalence}_{\text{Infected Syringes}}\right)^{m}$$

The force of infection for susceptible PWID participants in HR program is multiplied by a factor Z (Z<1) (2) indicating that PWID in HR programs have lower probability of getting infected compared to PWID not in HR programs (2).

After infection, there is a probability, C, that PWID will spontaneously clear the disease and are then at risk of re-infection. We conservatively assumed that the risk of re-infection is equal to that of initial infection. Those who do not clear the infection progress back to the chronically infected stage and will be eligible for re-treatment. The annual number of PWID to be treated is calculated by multiplying treatment rate (T) by the number of PWID in 2016. Every year, a fixed number of PWID are treated. If the number of chronic infections is lower than T, then all PWID receive treatment.

If PWID achieve SVR, then they become susceptible again and are at risk of re-infection, (assuming a risk of re-infection equal to the initial infection rate). In this case, the model allows the potential for changes in risk behavior after successful treatment. To account for this, the probability of infection is multiplied by a factor q, which represents the potential change in risk behavior. We assumed that the impact of the mental health counseling /education interventions during treatment results in 30% reduction of the re-infection probability, compared to a PWID receiving only treatment (3). If they did not achieve SVR, they return to the chronically infected component of the model. We choose to treat again the treatment failures as next-generation direct-acting antivirals (DAAs) are effective in first generation failing regimens (4).

The mean time to HCV acquisition was calculated by taking the inverse of the annual probability of infection. We assumed that only sharers could be infected and that the proportion of sharer and non-sharer PWIDs remained constant over time. Furthermore, potential natural immunity following successful treatment and transmission through sexual activity and non-injection drug use were not considered in the model.

4) Model calibration

The model was run until it achieved steady state, which is the level of HCV prevalence in the population of PWID in the absence of treatment, by varying the infection rate. Due to the small proportion of PWID who have been treated for CHC, we assumed that the treatment rate before 2016 was negligible. After reaching steady state, we examined intervention scenarios involving scaled-up treatment coverage and/or increased proportions of PWID on harm reduction programs and assessed their impact on CHC prevalence. We have also assessed under which intervention strategies HCV elimination is possible, which we define as reducing incidence by 80%.

The number of iterations was increased until further increases had no significant effect on the results (both in the central value but also in the confidence intervals). In our model, our results did not change when the number of iterations was more than 500.

5) Model parameterization and baseline incidence rate

The following table displays all the examined scenarios.

Table S1: Proportion of sharers and number of injections per person per year. The number of injections is computed by the model in order to achieve the target of chronic hepatitis C prevalence given the proportion of sharers.

Baseline chronic hepatitis C prevalence		
	Proportion of sharers (%)	Number of unsafe injections per person per year
30%	30 (low)	60
30%	50 (high)	37
45%	30	95
45%	50	50
60%	30	230
60%	50	80

Baseline annual HCV incidence rate

According to our model, HCV incidence rate in areas with 30% chronic HCV ranges 3.6-7.5 new infections per 100-person years. Similarly, under 45% or 60% baseline CHC prevalence, HCV incidence rate varies between 8.2-14 and 15.1-25.7 new infections per 100-person years, respectively.

Table S2: Real life settings for each examined scenario of the manuscript

**Setting**			**Reference**
	**CHC Prevalence (%)**	**Proportion of sharers (%)**	
Belgium	32	41	(5)
Switzerland	42	10	(6)
Hamburg Germany	50	25	(7, 8)
Italy	42	50	(9)
Norway	48	58	(10)
Portugal	64	20	(11)
Athens Greece	64	44	(12)
St. Petersburg Russia	66	41	(13)
