# Supplementary material for: HCV elimination among people who inject drugs. Modelling pre- and post–WHO elimination era
Source: PLoS One. 2018 Aug 16;13(8):e0202109. doi: 10.1371/journal.pone.0202109 (PMC6095544; doi:10.1371/journal.pone.0202109)
Supplement: S1 Table — The number of injections is computed by the model in order to achieve the target of chronic hepatitis C prevalence given the proportion of sharers. (PDF) [file pone.0202109.s002.pdf]

Supporting information

S1 Table. Proportion of sharers and number of injections per person per year. The number of injections is computed by the model in order to achieve the target of chronic hepatitis C prevalence given the proportion of sharers.

Baseline chronic hepatitis C prevalence		
	Proportion of sharers (%)	Number of unsafe injections per person per year
30%	30 (low)	60
30%	50 (high)	37
45%	30	95
45%	50	50
60%	30	230
60%	50	80
