# Supplementary material for: HCV elimination among people who inject drugs. Modelling pre- and post–WHO elimination era
Source: PLoS One. 2018 Aug 16;13(8):e0202109. doi: 10.1371/journal.pone.0202109 (PMC6095544; doi:10.1371/journal.pone.0202109)
Supplement: S2 Table — (PDF) [file pone.0202109.s003.pdf]

**Supporting information**

**S2 Table.** Real life settings for each examined scenario of the manuscript

| **Setting** | | |
|---|---|---|
| | **CHC Prevalence (%)** | **Proportion of sharers (%)** |
| Belgium | 32 | 41 |
| Switzerland | 42 | 10 |
| Hamburg Germany | 50 | 25 |
| Italy | 42 | 50 |
| Norway | 48 | 58 |
| Portugal | 64 | 20 |
| Athens Greece | 64 | 44 |
| St. Petersburg Russia | 66 | 41 |
