# Supplementary material for: HCV elimination among people who inject drugs. Modelling pre- and post–WHO elimination era
Source: PLoS One. 2018 Aug 16;13(8):e0202109. doi: 10.1371/journal.pone.0202109 (PMC6095544; doi:10.1371/journal.pone.0202109)

Supporting information

S1 Fig. Needed treatment coverage (%) to achieve HCV elimination by 2030 under a 30% chronic hepatitis C prevalence. The bars correspond to scenarios of harm reduction coverage at 40%, or 75% of PWID.

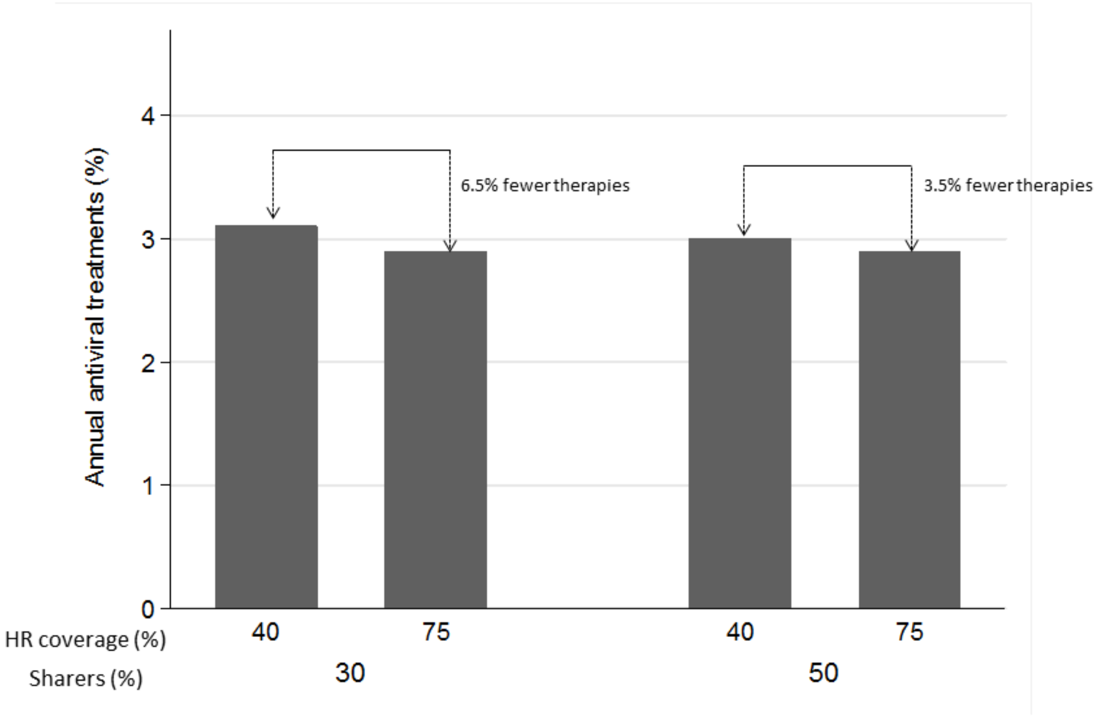

Supplement: S1 Fig — The bars correspond to scenarios of harm reduction coverage at 40%, or 75% of PWID. (PDF) [file pone.0202109.s005.pdf]
