# Supplementary material for: HCV elimination among people who inject drugs. Modelling pre- and post–WHO elimination era
Source: PLoS One. 2018 Aug 16;13(8):e0202109. doi: 10.1371/journal.pone.0202109 (PMC6095544; doi:10.1371/journal.pone.0202109)

Supporting Information

S3 Fig. Model predictions concerning a 45% chronic HCV prevalence in which 50% of the PWID are sharers. We assumed that no treatments are given after 2030. Mean time of HCV acquisition greater than 25 years is shown as 24+ years. Mean time corresponds to Tx only scenario. Tx: Antiviral treatment, HR: harm reduction, Counselling: Psychological interventions to reduce re-infections post- treatment.

A. Chronic HCV Prevalence

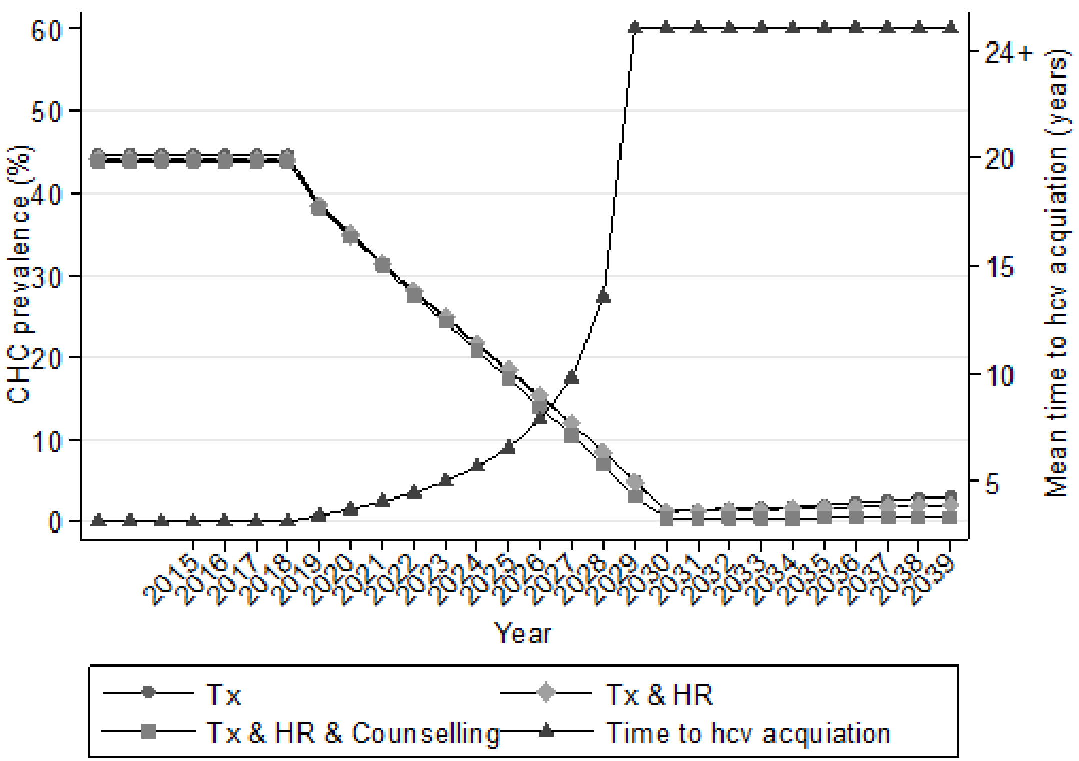

B. HCV Incident cases

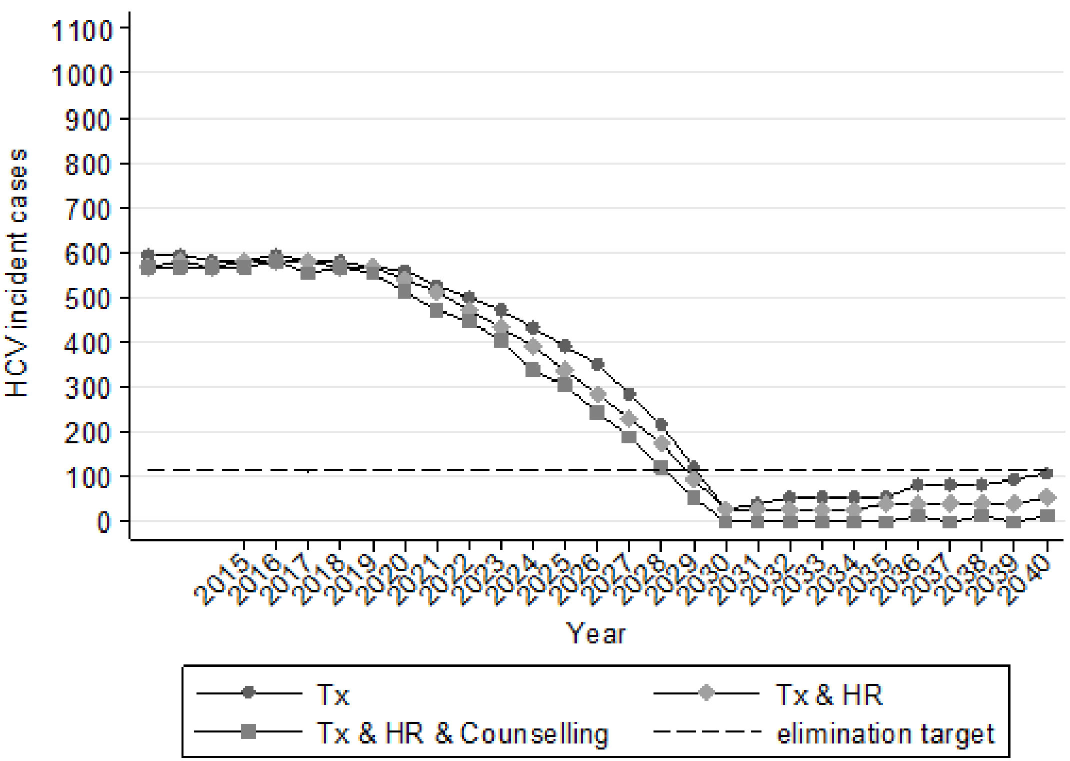

Supplement: S3 Fig — We assumed that no treatments are given after 2030. Mean time of HCV acquisition greater than 25 years is shown as 24+ years. Mean time corresponds to Tx only scenario. Tx: Antiviral treatment, HR: harm reduction, Counselling: Psychological interventions to reduce re-infections post- treatment. A. Chronic HCV Prevalence, B. HCV Incident cases. (PDF) [file pone.0202109.s007.pdf]
