# Supplementary material for: HCV elimination among people who inject drugs. Modelling pre- and post–WHO elimination era
Source: PLoS One. 2018 Aug 16;13(8):e0202109. doi: 10.1371/journal.pone.0202109 (PMC6095544; doi:10.1371/journal.pone.0202109)

Supporting Information

S4 Fig. Model predictions concerning a 60% chronic HCV prevalence and 50% of the people who inject drugs sharing injection equipment. Tx: Antiviral treatment, HR: harm reduction

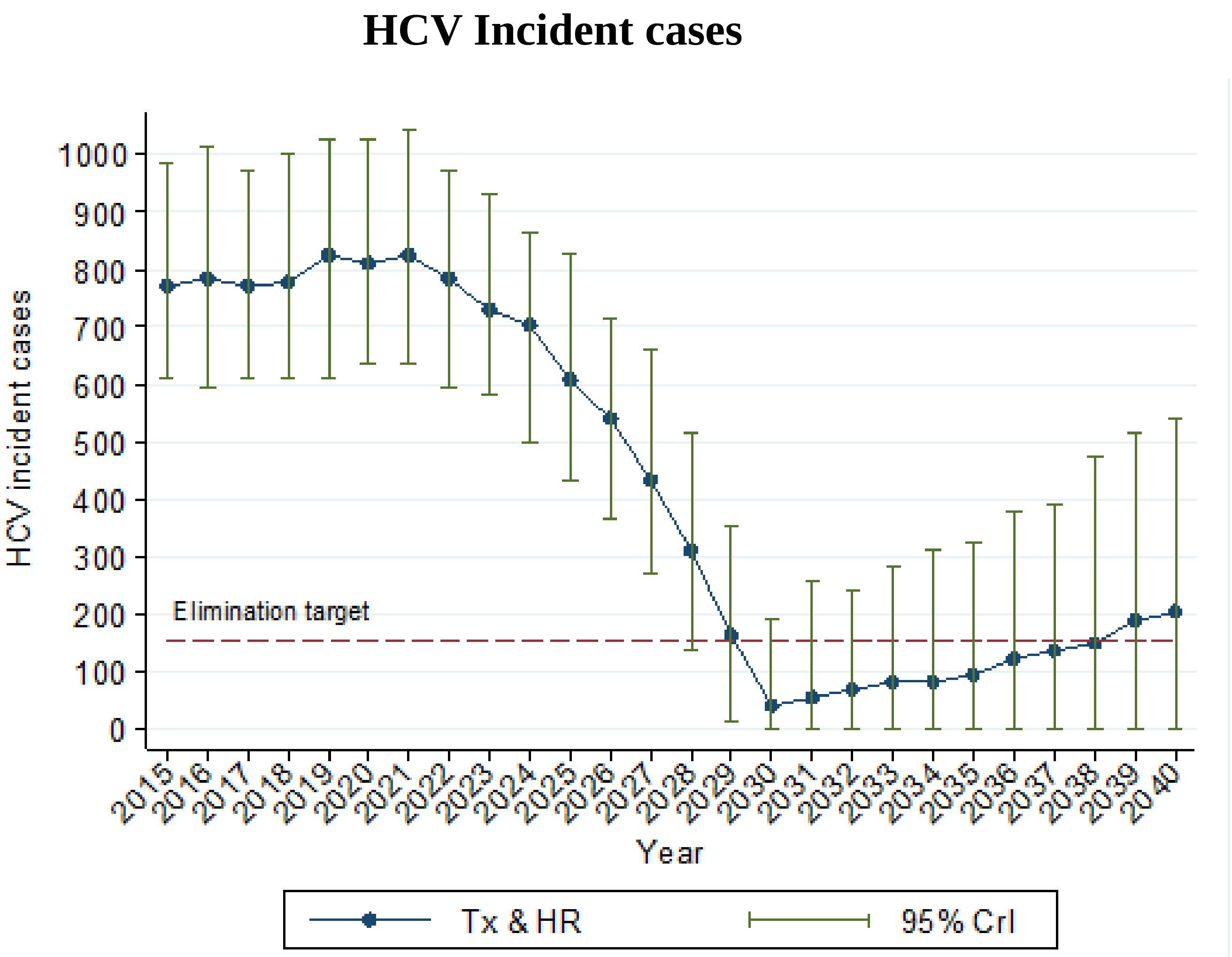

Supplement: S4 Fig — Tx: Antiviral treatment, HR: harm reduction. A. Sustainability of the elimination targets if incident cases reduced more than WHO elimination goals (e.g. 90% reduction in 2030 vs. to 80% reduction in 2030 compared to 2017). (PDF) [file pone.0202109.s008.pdf]
