# Supplementary material for: HCV elimination among people who inject drugs. Modelling pre- and post–WHO elimination era
Source: PLoS One. 2018 Aug 16;13(8):e0202109. doi: 10.1371/journal.pone.0202109 (PMC6095544; doi:10.1371/journal.pone.0202109)

Supporting Information

S5 Fig. Model predictions concerning a 45% chronic HCV prevalence in which 30% of the PWID are sharers. We assumed that no treatments are given after 2030. Tx: Antiviral treatment, HR: harm reduction.

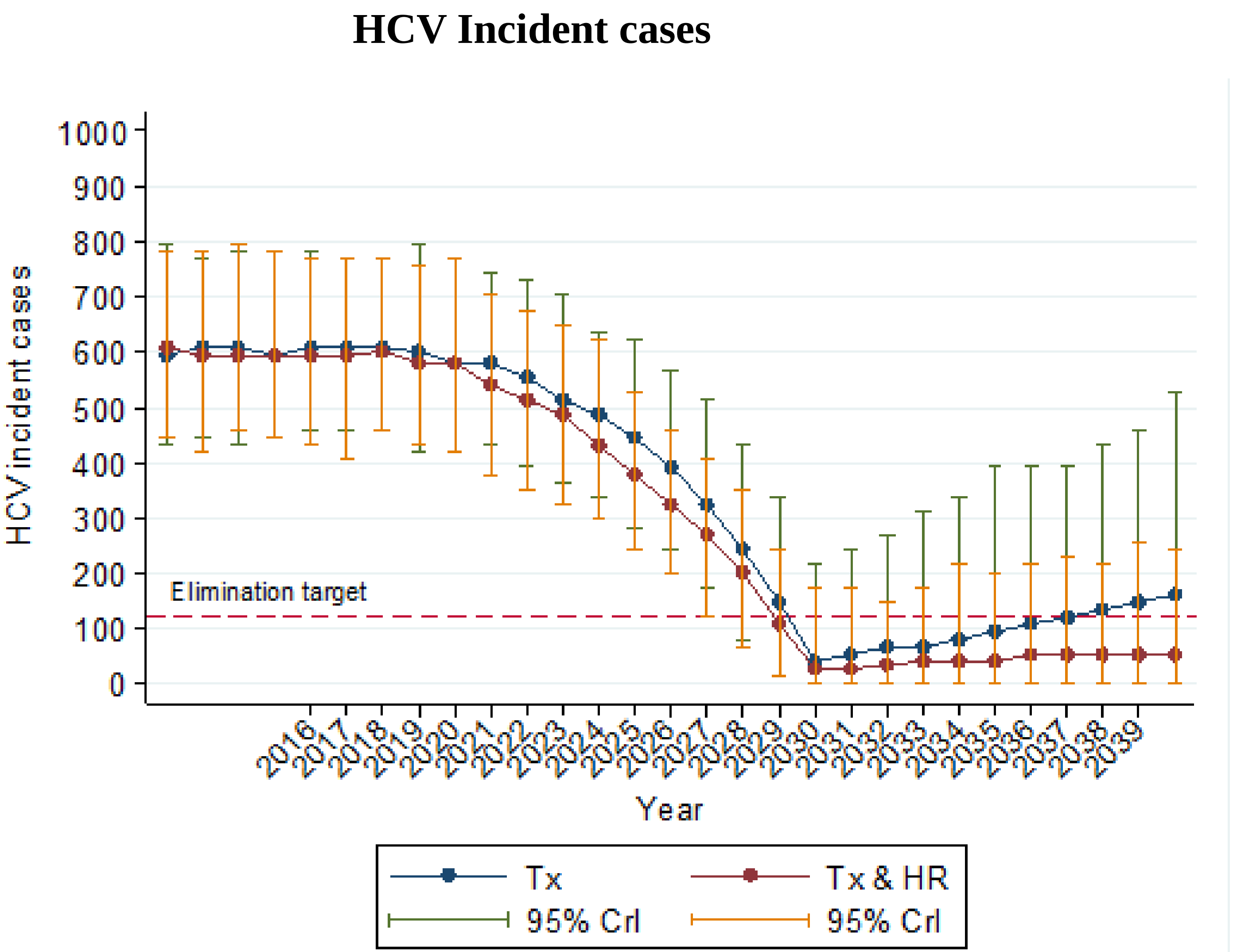

Supplement: S5 Fig — We assumed that no treatments are given after 2030. Tx: Antiviral treatment, HR: harm reduction. Model projection containing confidence intervals. (PDF) [file pone.0202109.s009.pdf]
