# Supplementary material for: HCV elimination among people who inject drugs. Modelling pre- and post–WHO elimination era
Source: PLoS One. 2018 Aug 16;13(8):e0202109. doi: 10.1371/journal.pone.0202109 (PMC6095544; doi:10.1371/journal.pone.0202109)

Supporting Information

S7 Fig. Needed treatment coverage (%) to achieve HCV elimination by 2030 under a 60% chronic hepatitis C prevalence with 20% or 40% baseline Harm reduction coverage.

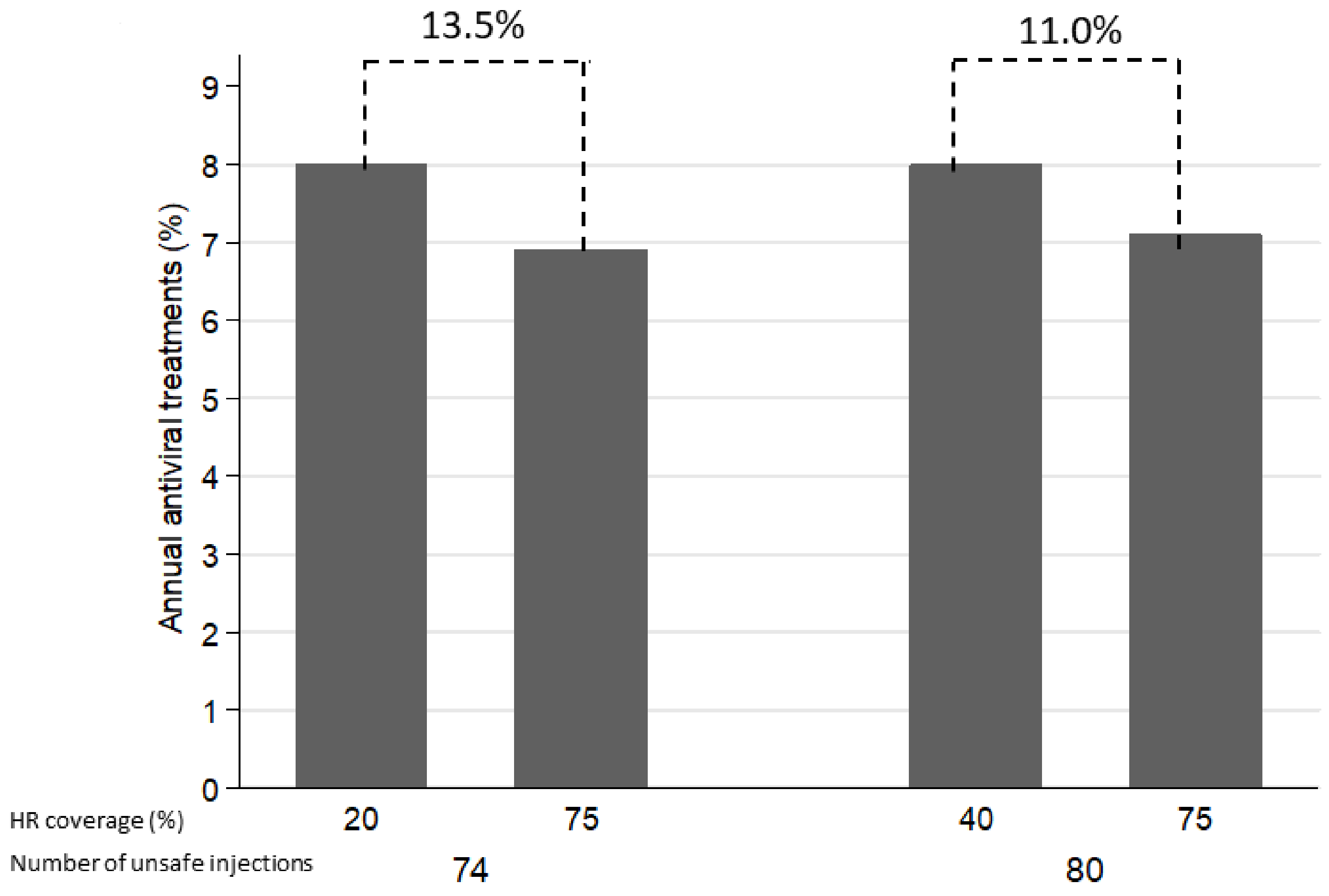

Supplement: S7 Fig — (PDF) [file pone.0202109.s011.pdf]
