# Supplementary material for: HCV elimination among people who inject drugs. Modelling pre- and post–WHO elimination era
Source: PLoS One. 2018 Aug 16;13(8):e0202109. doi: 10.1371/journal.pone.0202109 (PMC6095544; doi:10.1371/journal.pone.0202109)

Supporting Information

S8 Fig. Model predictions concerning a 45% chronic HCV prevalence in which 50% of the PWID are sharers. We assumed that treatments are reduced after 2020.

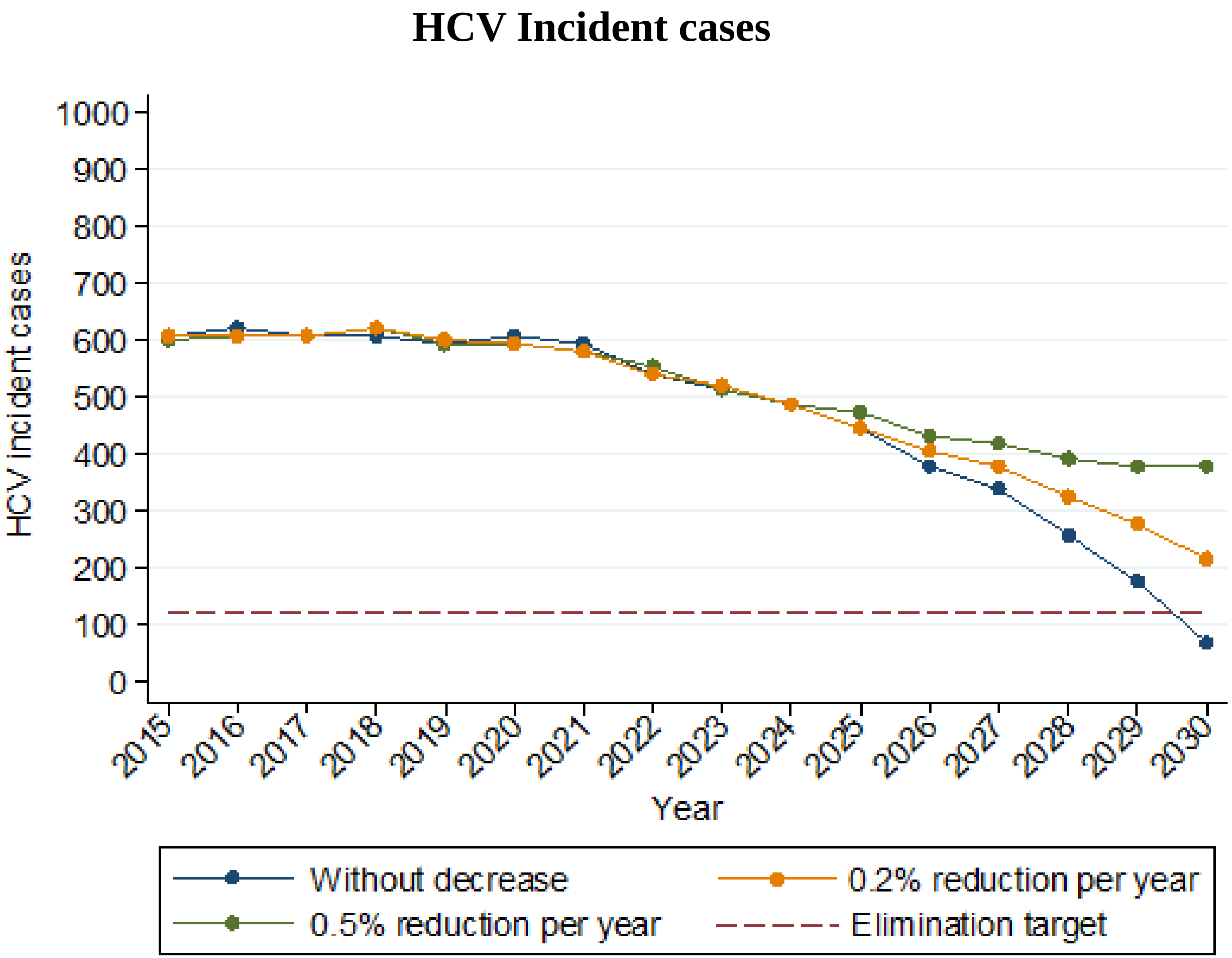

Supplement: S8 Fig — We assumed that treatments are reduced after 2020. Model projection assuming a decreasing of DAAs coverage prior to 2030 as a result of as a result of the unsuccessful the implementation of awareness or screening campaigns. (PDF) [file pone.0202109.s012.pdf]
